# Electrochemical Detection and Analysis of Various Current Responses of a Single Ag Nanoparticle Collision in an Alkaline Electrolyte Solution

**DOI:** 10.3390/ijms23137472

**Published:** 2022-07-05

**Authors:** Ki Jun Kim, Seong Jung Kwon

**Affiliations:** Department of Chemistry, Konkuk University, 120 Neungdong-ro, Gwangjin-gu, Seoul 05029, Korea; kim573252@naver.com

**Keywords:** silver (Ag), single nanoparticle, electrocatalytic amplification, electrochemistry, single-molecule studies, alkaline solution

## Abstract

A single silver (Ag) nanoparticle (NP) collision was observed and analyzed in an alkaline solution using the electrocatalytic amplification (EA) method. Previously, the observation of a single Ag NP collision was only possible through limited methods based on a self-oxidation of Ag NPs or a blocking strategy. However, it is difficult to characterize the electrocatalytic activity of Ag NPs at a single NP level using a method based on the self-oxidation of Ag NPs. When using a blocking strategy, size analysis is difficult owing to the edge effect in the current signal. The fast oxidative dissolution of Ag NPs has been a problem for observing the staircase response of a single Ag NP collision signal using the EA method. In alkaline electrolyte conditions, Ag oxides are stable, and the oxidative dissolution of Ag NPs is sluggish. Therefore, in this study, the enhanced magnitude and frequency of the current response for single Ag NP collisions were obtained using the EA method in an alkaline electrolyte solution. The peak height and frequency of single Ag NP collisions were analyzed and compared with the theoretical estimation.

## 1. Introduction

Recently, metal nanoparticles (NPs) have been widely used in many fields owing to their large surface-to-volume ratio and size-dependent optical properties [1,2,3,4]. The study of the electrocatalytic or other properties of NPs at a single NP level is challenging because of the difficulty in identifying a small signal among noisy signals. Therefore, most research on NPs has been based on ensemble-averaged properties of many NPs [5,6]. However, the study of NPs at a single NP level offers several advantages, including the detection of reactive intermediates [7] and the discovery of rare events that cannot be observed by traditional ensemble-based electrochemical methods [8]. A deeper understanding of the behavior of NPs at a single NP level can provide clues for a novel nanostructure design in many applications, such as nano-catalysts, nano-electronics, and nano-devices [9,10].

In recent years, electrochemical methods for detecting single-NP collisions on electrode surfaces have been studied. In this technique, when an NP collides with the electrode surface, the collision is observed as a current or potential signal according to its electrocatalytic amplification (EA). This method is called a single NP collision method or nano-entity electrochemistry [11,12,13,14,15,16,17,18].

When NPs collide with an electrode, the collision produces an individual signal. This process is stochastic. The NP size and concentration are proportional to the magnitude and frequency of the signal, respectively [13,19]. Therefore, a simple analysis of the current signal can provide information regarding the concentration and size distribution of the NP. In addition, the individual electrocatalytic properties of single NPs can be obtained based on an analysis of the shape or durability of individual signals. The catalytic reaction mechanism of NPs can also be proposed based on the characteristics of current response, such as staircase [13], blip [15], spike [17], and reverse cases [20].

Such observations and analyses of single-NP collisions have been applied to earlier studies of hard particles, such as NPs of metals or metal oxides (e.g., Pt [13,14], IrO_x_ [15], Ag [16], Au [21], Cu [17,22], and TiO_2_ [23]), or nonconducting particles such as polystyrene or latex [20]. Not only hard particles, but also soft NPs such as emulsions [24,25] and biomaterials such as viruses, proteins, and DNA [26,27], can be used for single NP observation. Target nanomaterials that can be explored are expanding with the advancement of this technique.

Ag NPs are among the extensively studied NPs. Ag NPs are widely used in antimicrobial coatings for industrial products because they are well known for their antibacterial effects [28]. Ag NPs are also used as labels to introduce thiol self-assembled monolayers in various bio-applications [29].

The single-NP collision method has also been applied to the analysis of Ag NPs. In previous studies, a single Ag NP collision signal was observed based on the self-oxidation of Ag NPs [10,30,31,32] or by the blockage of the electrocatalytic reaction at the ultramicroelectrode (UME) by a Ag NP, i.e., a blocking strategy [33]. The conventional single NP collision using the EA method were difficult to apply to Ag NPs because Ag is not a good electrocatalyst compared to commonly used alternatives such as Au or Pt. Therefore, single Ag NP collisions were achieved only in a limited condition. This is because the detection of Ag NPs based on self-oxidation or the blocking strategy requires a significantly large NP size of at least sub-micrometers. Therefore, the observation of single Ag NP collision using the EA method is advantageous in sensitivity compared to recent studies on single Ag NP collision using a blocking strategy or self-oxidation [32,34,35]. This has helped to avoid the problem in which a signal could not be observed when the NPs were small [12,20].

In this study, to improve the sensitivity of single Ag NP collisions, a single Ag NP based on the EA method was obtained using an alkaline electrolyte solution for the hydrazine oxidation reaction. In an alkaline electrolyte solution, Ag is easily oxidized to AgO, which is stable and exhibits good electrocatalytic activity for hydrazine oxidation [36]. Based on the electrocatalytic activity of the oxidized Ag NP, a staircase current response was obtained for the first time in Ag NP collisions. The oxidation state of Ag NP according to the applied potential and its electrocatalytic activity for the hydrazine oxidation were also investigated.

## 2. Results and Discussion

**Figure 1:**
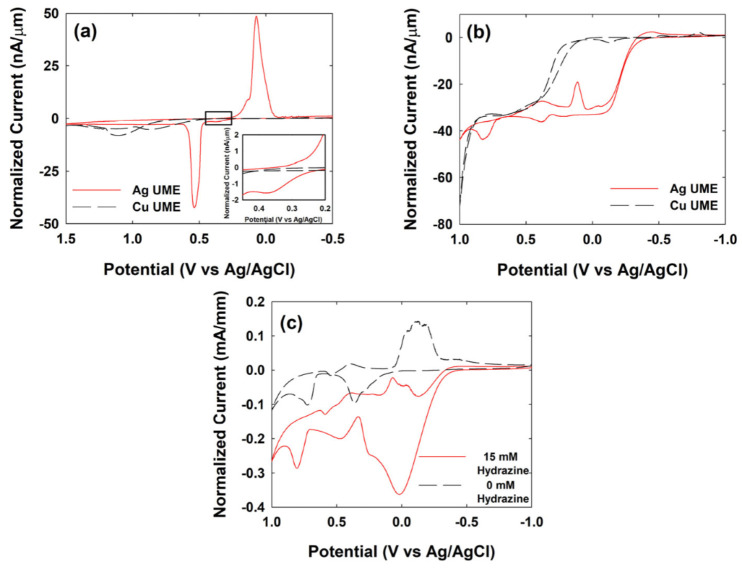
Cyclic voltammograms of Ag UME (red solid) and Cu UME (black dashed) in (**a**) 50 mM PB or (**b**) 0.1 M NaOH solution containing 15 mM hydrazine. The current was normalized based on the radius of UMEs (radius: 7.5 μm for Cu and 12.5 μm for Ag). (**c**) Cyclic voltammograms of Ag NP modified GCE in 0.1 M NaOH with (red solid) and without (black dashed) 15 mM hydrazine. The diameter of GCE is 3 mm. The scan rate was 0.1 V/s.

The electrocatalytic characteristics of Ag and Cu for hydrazine oxidation depending on the pH of the electrolyte solution were investigated using cyclic voltammetry (CV). As shown in Figure 1, the hydrazine oxidation at the Ag and Cu UME were tested in a 50 mM phosphate buffer (PB) or 0.1 M NaOH. Because Ag NPs are surrounded by surfactants such as citrate, the electrocatalytic behavior could be slightly different from that of the bare Ag UME. Therefore, glassy carbon electrodes (GCEs) modified with Ag NPs were also prepared, and their electrocatalytic activity for hydrazine oxidation was investigated.

**Figure 2:**
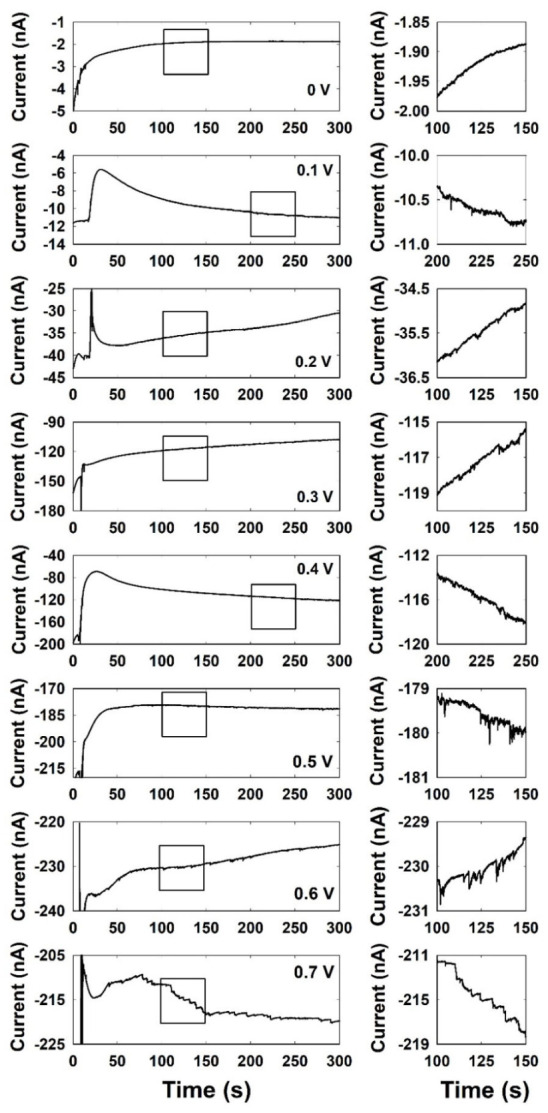
Chronoamperometric curves for single Ag NP collisions at the Cu UME with different applied potentials from 0 V to 0.7 V in a 0.1 M NaOH solution containing 15 mM hydrazine. Data acquisition time is 50 ms.

Under neutral pH conditions, both Ag and Cu UMEs did not show any considerable catalytic characteristics for hydrazine oxidation (Figure 1a). In the case of the Ag UME, a small current was produced at ~0.35 V (vs. Ag/AgCl), indicating electrocatalytic hydrazine oxidation. The Ag UME also had a strong oxidation peak at ~0.53 V and a reduction peak at ~0.07 V, which were related to the redox reaction of the Ag itself. In the oxidation scan, the current after the oxidation of Ag was maintained at a low level (~2 nA/μm), indicating a low electrocatalytic reaction rate. This is because the Ag oxide also has low electrocatalytic activity for hydrazine oxidation in neutral pH [37]. Conversely, the Cu UME showed small and wide oxidation currents at a potential range from ~0.5 V to ~1.2 V, which indicated the oxidation of the Cu. As shown in Figure 2a, the electrocatalytic activity of Ag for hydrazine oxidation at neutral pH is negligible; therefore, the blocking strategy has mainly been used for single Ag NP detection in previous studies.

However, in the alkaline condition, both Ag and Cu UMEs showed an electrocatalytic current for the hydrazine oxidation reaction at −0.40 V and 0.20 V, respectively (Figure 1b and Figure S1, see the Supplementary Materials). The Ag UME had a lower onset potential than the Cu UME.

Unlike in the PB, the Ag UME had two oxidation peaks at ~0.40 V and ~0.72 V, and their corresponding reduction peaks at 0.38 V and 0.07 V in 0.1 M NaOH (Figure 1b). It seems that the first oxidation peak at ~0.40 V corresponds to oxidation from Ag (0) to Ag (I), and the second oxidation peak ~0.72 V corresponds to the oxidation of Ag (I) to Ag (II) [36]. As shown here, the Ag UME has a clear two-step oxidation and a steady-state current was maintained after the oxidation of Ag. This indicates that the Ag electrode surface in alkaline electrolyte conditions exhibits electrocatalytic activity for hydrazine oxidation, which is maintained even after the phase change from Ag to Ag oxide.

As shown in Figure 1c, the GCE modified with Ag NPs also showed two similar oxidation peaks at ~0.45 V and ~0.77 V in 0.1 M NaOH. The electrocatalytic activity for hydrazine oxidation was maintained even after the Ag NPs were oxidized in the higher-potential region.

For the detection of a single NP collision with the EA method, the material with a relatively lower electrocatalytic activity was used as the UME, and that with higher activity was used as an NP form. Therefore, the Ag NP collision on the Cu UME was investigated in a 0.1 M NaOH solution in the presence of hydrazine. The single Ag NP collision response varied with the applied potential.

Here, the concentration of electrolyte is optimized for the detection of single NP’s collision (Figure S2). When the concentration of electrolyte is too low (<~10 mM), the increased solution resistance affects to the electrocatalytic reaction and measurement of the response. When the concentration of electrolyte is too high (>~1 M), side effects, such as aggregation of NPs and formation of various hydroxide complex, are expected. Therefore, the 0.1 M of NaOH concentration was selected to ensure same electrolyte level with the case of buffer solution. An effect on the current response by residues such as citrate or KNO_3_ in the NP stock solution was also investigated. As showed in Figure S3, the citrate and KNO_3_ did not show any electrochemical signal.

As shown in Figure 2, a single Ag NP collision was recorded via the chronoamperometric method at various potentials applied to the Cu UME. The Ag NP collision signals were observed very rarely in the potential ranges from 0.0 V to 0.4 V. However, the current transient was observed as a blip response at 0.5 and 0.6 V and a staircase response at 0.7 V. It is worth noting that the current responses were clearly observed in a potential region higher than the potential at which the Ag is oxidized, implying that the oxidation of Ag and the signal observation are related.

The change in current response depending on the applied potential seems to be due to the difference in the collision mechanism and oxidation state of the Ag NP. According to the results of typical single NP collision studies, the current signal by single NP collisions should be obtained at potential ranges from 0 V to 0.4 V, in which the electrocatalytic activities of Ag and Cu are different. However, as shown in Figure 2, a single Ag NP collision is rarely observed in this potential range. The very low collision frequencies at potentials below 0.4 V are thought to be due to the non-adhesive collision of Ag NP with a Cu UME.

Previous studies have shown that the signal response of a single NP collision can vary depending on its collision mechanism [15,22,33]. The collision mechanism of a single NP collision can be classified as hit-n-run, hit-n-roll, or hit-n-stay, depending on the state of the NP after the collision event [7]. In particular, when Cu was used as a UME to observe the single-NP collision signals of Pt NPs and IrO_x_ NPs, it was observed that the collision frequency was significantly reduced to ~1/3000 for Pt NPs and ~1/4 for IrO_x_ NPs compared to the other electrode materials [38,39]. Therefore, we believe that the reduced frequencies are because of the weak interaction between the NPs and Cu UME, which leads to a hit-n-run mechanism in which the colliding Ag NP does not adhere well to the Cu UME. This is discussed in a later section.

However, when the applied potential to the Cu UME is increased to over 0.5 V, the oxidation of Ag NP occurs.

At a potential region of 0.5 and 0.6 V, Ag (0) is oxidized to Ag (I) [36].

$$2\mathrm{Ag}+2{\mathrm{OH}}^{-}\;↔{\mathrm{Ag}}_{2}O+H_{2}O+2e^{-}$$

When a voltage above 0.7 V was applied, Ag (0) was oxidized to Ag (II) [36].

$$\mathrm{Ag}+2{\mathrm{OH}}^{-}↔\mathrm{AgO}+H_{2}O+2e^{-}$$

The magnitude and frequency of the current responses of a single Ag NP in this potential region were further increased. These magnitudes and frequencies of the current signals at various applied potentials are specified in Table 1. The observation of distinguishable blip and staircase current responses in this potential region compared to the small and rare responses at a low potential region of less than 0.4 V can be explained based on several factors.

First, the high electrocatalytic activity of Ag oxide was responsible for the enhanced signals. Many previous studies have reported high electrochemical activity of Ag (I and II) oxide for hydrazine oxidation [40,41]. When Ag oxide powder is immersed directly in the hydrazine solution, it reacts explosively [33]. Accordingly, the oxidation of Ag NPs in this potential region may accelerate the electrocatalytic hydrazine oxidation, thereby increasing the magnitude of the collision signal.

In this potential region, both the Ag NP and Cu UME are oxidized. The changes in the surfaces of both the NP and UME can affect the interaction between the NP and UME at the moment of collision. Crosslinking between Ag and Cu by oxygen atoms is possible during the vigorous oxidation of Ag NPs on the oxidized Cu UME [42]. This can render the collision between the Ag NP and Cu UME more adhesive, and the collision mechanism tends to be close to hit-n-stay.

The change in the collision mechanism from hit-n-run to hit-n-stay according to the change in the Cu UME and Ag NP surface can be confirmed by investigating the remaining Ag NPs after single NP collision experiments. If Ag NPs remain on the electrode surface after the collision event, the electrocatalytic current of the residual Ag NP can be observed by cyclic voltammetry measurements after a single NP collision. As shown in Figure S4, when cyclic voltammetry was performed after a single Ag NP collision experiment at 0.4 V and 0.7 V, respectively, the electrocatalytic current by the remaining Ag NPs was obtained only when 0.7 V was applied. This could be evidence that the hit-n-run collision mechanism occurs at a potential of 0.4 V or less where the Ag NP is not oxidized and that the hit-n-stay collision mechanism occurs at a potential of 0.5 V or more where the Ag NP is oxidized.

The migration effect is another reason for the increased collision frequency. The Ag NP surrounded by citrate ions was negatively charged. Therefore, the increased positive (oxidative) potential on the electrode accelerates the migration of Ag NP to the Cu UME [43,44].

Lastly, the different current responses, blip responses at 0.5 V and 0.6 V and staircase response at 0.7 V, can be explained by the different reaction rates of the oxidative dissolution of Ag NPs. Oxidative dissolution of Ag NPs in the solution occurs when metallic Ag is oxidized. When a single Ag NP collision was investigated under neutral pH conditions in a previous study [33,45], as mentioned above, the Ag NP had negligible electrocatalytic activity for hydrazine oxidation at neutral pH; thus, a decrease in the electrocatalytic current was obtained by the blocking strategy whenever the Ag NPs collided with the active electrocatalytic Au UME. When the applied potential is increased until the Ag NPs are oxidized, a spike-like electrocatalytic current is observed. This is due to the decomposition of the collided Ag NP by the oxidative dissolution process at neutral pH. In a previous study, the oxidative dissolution of Ag NPs during collision experiments at neutral pH has been reported in the form of multi-peak behavior that continues to decrease in size [45].

However, the oxidative dissolution of Ag NPs is much slower in alkaline solutions because the stability of the Ag oxide at alkaline pH is better than that at a neutral pH [36]. Therefore, the current decay due to oxidative dissolution is slow under alkaline conditions, and the current response changes from a spike to blip to staircase. The spike shape of current responses was obtained in a previous study under neutral pH conditions. In alkaline conditions, the blip response at 0.5 V and 0.6 V and the staircase response at 0.7 V were obtained as shown in Figure 2.

**Figure 3:**
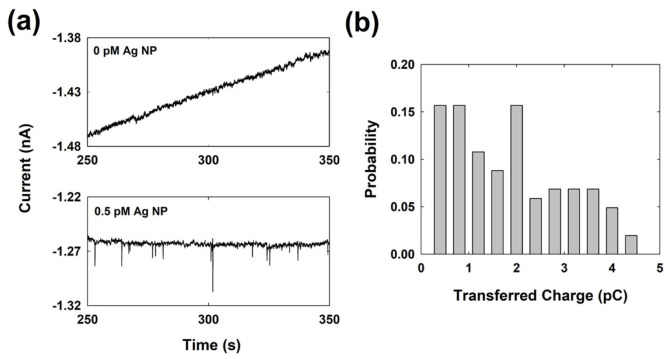
(**a**) Chronoamperometric curves at 0.7 V applied to a Cu UME in a 0.1 M NaOH solution with/without Ag NPs in the absence of hydrazine. (**b**) Transferred charge distribution of a spike like current response for a single Ag NP collision in the absence of hydrazine. The average transferred charge is 1.8 ± 1.1 pC.

To confirm that the staircase current response was due to electrocatalytic hydrazine oxidation by the Ag oxide NPs, a single Ag NP collision was investigated at 0.7 V in the absence of hydrazine. As shown in Figure 3, small spike-like current responses were observed. The average transferred charge for a single spike of transient current was 1.8 ± 1.1 pC, which is similar to the theoretical expected value of 1.23 pC (which is calculated based on the oxidation of an average-sized Ag NP).

The experimental results indicate that the spike-like current response are caused by the self-oxidation of Ag NPs and that electrocatalytic hydrazine oxidation is responsible for the staircase current response at 0.7 V.

Unlike the blip response, the staircase response obtained using the EA method can be analyzed theoretically in terms of the peak height and frequency. The staircase current step at 0.7 V, that is, the peak height, was analyzed and compared with the estimated value based on the theoretical calculation. The theoretical amplified current step can be calculated using the following equation [19,46]:

$$I_{SS,NP}=4\pi \left(ln2\right)nFD_{N2H4}C_{N2H4}r_{NP}$$

where *n* (=4) is the number of electrons required for hydrazine oxidation, *F* is the Faraday coefficient, *D*_N2H4_ is the diffusion coefficient of hydrazine, *C*_N2H4_ is the concentration of hydrazine, and *r*_NP_ is the radius of the Ag NP. Here, the diffusion coefficient of hydrazine was estimated using the steady-state current of the Ag UME shown in Figure 1b using the following equation: [19,46]

$$I_{\mathrm{SS},\mathrm{UME}}=4nFD_{N2H4}C_{N2H4}r_{\mathrm{UME}}$$

where *r*_UME_ is the radius of the Cu UME. The calculated theoretical peak height is 1.9 nA. This value is higher than 0.23 ± 0.16 nA, which is an experimental value (as shown in Table 1 and Figure S5). The general calculation of peak height by EA method is based on the case where the hydrazine does not react at all in UME, but only at the NP. However, in this experiment, the potential was increased to 0.7 V, at which point the hydrazine oxidation occurred even in the Cu UME. Therefore, for accurate calculations, the *C*_N2H4_ in the calculation equation should be revised by the effective concentration of hydrazine that actually arrived at the NP, excluding the amount consumed by the Cu UME. Here, the consumption of hydrazine by the Cu UME reduces the effective concentration of hydrazine, thereby reducing the peak height of current signal.

**Figure 4:**
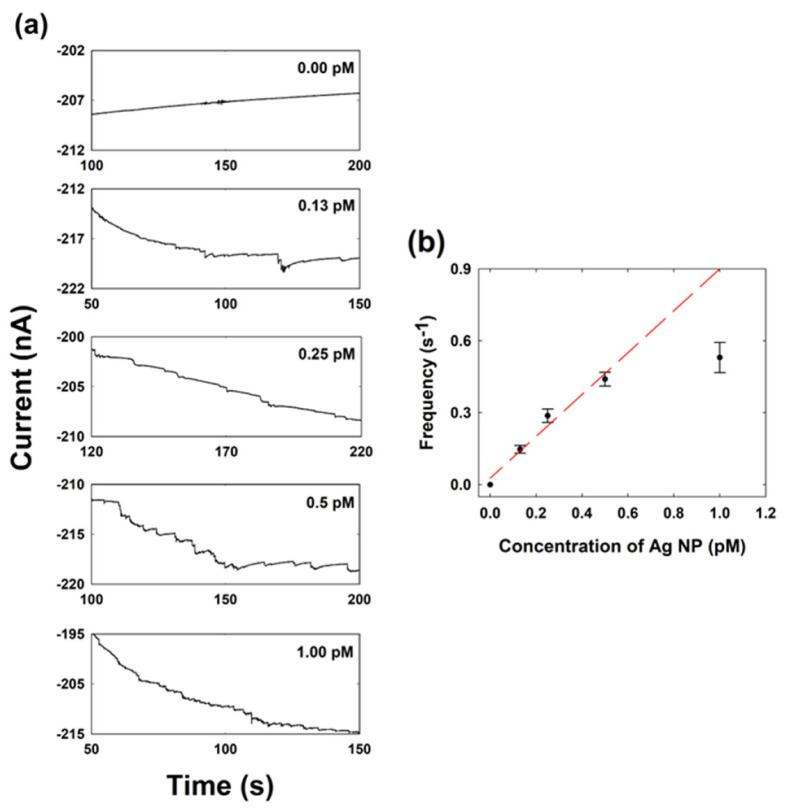
(**a**) Chronoamperometric curves of single Ag NP collisions obtained when 0.7 V is applied to a Cu UME with different concentrations (0 pM to 1.00 pM) of Ag NP in a 0.1 M NaOH solution containing 15 mM hydrazine. (**b**) Correlation plot between the collisional frequency and the concentration of the Ag NP.

The change in the collisional frequency of the single Ag NP collision depending on the NP concentration was also investigated at 0.7 V. As shown in Figure 4, the collisional frequency was linearly proportional to the Ag NP concentration in the low-concentration region. The theoretical collision frequency was calculated using the following equation: [19,46]

$$f_{p}=4D_{\mathrm{NP}}C_{\mathrm{NP}}r_{\mathrm{UME}}$$

where *D*_NP_ is the diffusion coefficient of Ag NPs estimated based on the Stokes–Einstein equation and *C*_NP_ is the concentration of Ag NP. The calculated value was 0.18 s^−1^ pM^−1^, but the experimental frequency (slope in the plot) was 0.87 s^−1^ pM^−1^. The calculated frequency was lower than the experimental frequency. This frequency calculation relies only on the diffusion of NPs without considering the migration. However, at 0.7 V, an additional mass transfer of NPs by the migration effects will occur due to the large positive potential, resulting in an increase in the collision frequency over the calculated values.

Given peak height and frequency analysis, it is reasonable to consider that the current response of NP is mainly due to a single NP, not an aggregate of NPs.

The current response and electrolyte conditions in various studies about Ag NP collision are summarized in the Table 2. Compared to the blip response of single Ag NP collision based on the self-oxidation of Ag NP itself, the staircase response using the EA method has relatively larger collisional frequency even when the applied potential is low. The reason for the increase in frequency is that the small NPs, which could not be observed using self-oxidation method, can be observed using the EA method. Therefore, it is possible to easily distinguish the current responses of the Ag NP collisions from noise current by using the NaOH electrolyte.

## 3. Methods and Materials

### 3.1. Chemicals

Silver nitrate (AgNO_3_), sodium citrate tribasic dehydrate (C_6_H_5_Na_2_O_7_·2H_2_O), hydrazine, and all buffer salts were obtained from Sigma or Aldrich (St. Louis, MO, USA), unless otherwise stated. Sodium hydroxide (NaOH) was obtained from Junsei (Tokyo, Japan). All chemicals were used as received. Ultrapure water (>18 MΩ, Millipore) was used in all the experiments. Ag (99.99%, diameter of 25 μm) wires were obtained from Goodfellow (Devon, PA, USA) and Cu (99.99%, diameter of 15 μm) wires were obtained from Alfa Aesar (Ward Hill, MA, USA). All the metal wires were used to fabricate the ultramicroelectrode (UME).

### 3.2. Preparation of Metal Nanoparticles (NPs)

**Figure 5:**
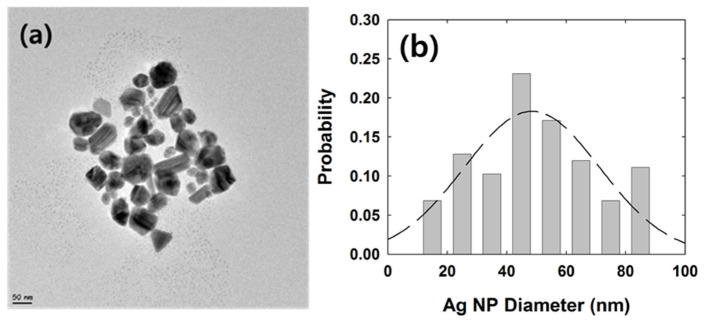
(**a**) TEM image of Ag NPs. (**b**) Size distribution of Ag NPs in TEM image. The average diameter is 50 ± 19 nm. The average surface area of NP is 6.5 × 10^4^ nm^3^.

Ag NPs were prepared by the reduction of the Ag precursor (AgNO_3_) with sodium citrate tribasic dihydrate [47]. AgNO_3_ (1 mM, 18 mL) was heated until it began to boil. Sodium citrate (1 mM, 2 mL) was added dropwise to the AgNO_3_ solution as soon as boiling commenced. The color of the solution slowly turned grayish yellow, indicating a reduction of the Ag^+^ ions. The solution was then stirred for 30 min to stop the heating. The sizes of the synthesized NPs were analyzed by transmission electron microscopy (TEM) and dynamic light scattering (DLS) measurement. The sizes of the Ag NPs analyzed by TEM and DLS were 50 ± 19 nm and 59 ± 25 nm, respectively, as shown in Figure 5 and Figure S6. NP concentration was calculated as the concentration of the Ag precursor divided by the number of Ag atoms in a NP. The calculated stock concentration of the Ag NP was 260 pM. The XRD data of synthesized Ag NP was investigated to figure out the phase structure of Ag NP (Figure S7).

### 3.3. Preparation of the UME

UMEs (diameter: 15 μm for Cu and 25 μm for Ag) were prepared with each metal wire sealed in borosilicate glass, according to a previous report [13,14,15]. The metal wire was connected to an electric wire using silver epoxy. Then, the electrode was polished until the metal wire was exposed. All the electrodes were polished to obtain a mirror surface using microcloth pads (Buehler, Lake Bluff, IL, USA).

### 3.4. Instrumentation

Electrochemical experiments were performed using a CHI 750e potentiostat (CH Instruments, Austin, TX, USA) with a three-electrode cell in a Faraday cage. The electrochemical cell consisted of a UME (working electrode), Pt wire counter electrode, and Ag/AgCl reference electrode. TEM images were obtained using a Tecnai G2 F30ST (FEI. Company, Hillsboro, OR, USA) at the Korea Basic Science Institute (Seoul, Korea). DLS analysis was performed using a Zetasizer Nano ZS90 instrument (Malvern, Worcestershire, UK).

### 3.5. Electrochemical Cell and Technique

All experiments were conducted in a three-electrode cell. A 50 mM phosphate buffer (PB) or NaOH solution containing hydrazine were used as an electrolyte solution. The chronoamperometric measurements were obtained using 50 ms of sampling time. The Ag NPs were injected into the electrolyte solution after the measurement was started.

## 4. Conclusions

Single Ag NP collisions were investigated in an alkaline solution by the electrocatalytic amplification (EA) method. The transient signals varied depending on the applied potential. The blip response was observed at applied potentials of 0.5 and 0.6 V. A staircase response was observed at 0.7 V. The enhanced magnitude and frequency of the current response are responsible for the oxidation of the Ag NP, increased migration speed, and slow oxidative dissolution of the Ag NP under alkaline conditions. The peak height and frequency of single Ag NP collisions were analyzed and compared with the theoretical calculation.

Therefore, the observation and analysis of a single Ag NP by the EA method were successfully carried out in an alkaline electrolyte solution. This method can be used for more accurate characterization and analysis of Ag NPs at a single NP level compared to a blocking strategy, meaning that it can be utilized in various applications of Ag NPs, for example, for improvement of electrocatalytic activity and stability of Ag NP or detection of Ag NP in complex environmental conditions.

## Figures and Tables

**Table 1:** Analysis of the current signal of a single Ag NP collision.

Potential (V)	Signal Type	Peak Height (nA)	Transferred Charge (pC)	Frequency (s^−1^ pM^−1^)
0	-	-	-	-
0.1	Spike	0.078 (±0.032)	10 (±6)	0.06
0.2	Spike	0.074 (±0.022)	9 (±4)	0.14
0.3	Spike	0.30 (±0.10)	23 (±9)	0.18
0.4	Spike	0.28 (±0.12)	25 (±11)	0.13
0.5	Blip	0.32 (±0.15)	30 (±20)	0.28
0.6	Blip	0.28 (±0.15)	70 (±60)	0.48
0.7	Staircase	0.23 (±0.16)	-	0.87

**Table 2:** Comparison of signal frequency and peak height of current response of a single Ag NP collision.

Response Type	Applied Potential (V) ^c^	Frequency (s^−1^ pM^−1^)	Electrolyte	Reference
Spike ^a^	0.5	0.04	10 mM citrate & 90 mM KCl	[16]
Spike ^a^	0.6	0.016	10 mM Na_2_S_2_O_3_ & 10 mM NaOH	[32]
Blip ^b^	1.3	1.0	50 mM PB	[34]
Spike ^a^	0.5	0.16	10 mM NaNO_3_ & 10 mM PB	[35]
Spike ^a^	1.65	~0.013	50 mM KNO_3_	[36]
Staircase ^b^	0.7	0.87	0.1 M NaOH	This work

^a^ By self-redox reaction; ^b^ by hydrazine oxidation; ^c^ vs. Ag/AgCl reference electrode.

## Data Availability

Not applicabe.

## Supplementary Materials

The following supporting information can be downloaded at: https://www.mdpi.com/article/10.3390/ijms23137472/s1.

## References

[1] Stark W.J., Stoessel P.R., Wohlleben W., Hafner A.J.C.S.R. (2015). Industrial applications of nanoparticles. Chem. Soc. Rev..

[2] Van Broekhuizen P., van Broekhuizen F., Cornelissen R., Reijnders L. (2011). Use of nanomaterials in the European construction industry and some occupational health aspects thereof. J. Nanoparticle Res..

[3] Bauer L.A., Birenbaum N.S., Meyer G.J. (2004). Biological applications of high aspect ratio nanoparticles. J. Mater. Chem..

[4] Liebel M., Calderon I., Pazos-Perez N., van Hulst N.F., Alvarez-Puebla R.A. (2022). Widefield SERS for High-Throughput Nanoparticle Screening. Angew. Chem. Int. Ed..

[5] Chen J., Lim B., Lee E.P., Xia Y. (2009). Shape-controlled synthesis of platinum nanocrystals for catalytic and electrocatalytic applications. Nano Today.

[6] Spendelow J.S., Wieckowski A. (2007). Electrocatalysis of oxygen reduction and small alcohol oxidation in alkaline media. Phys. Chem. Chem. Phys..

[7] Kong N., Guo J., Chang S., Pan J., Wang J., Zhou J., Liu J., Zhou H., Pfeffer F.M., Liu J. (2021). Direct observation of amide bond formation in a plasmonic nanocavity triggered by single nanoparticle collisions. J. Am. Chem. Soc..

[8] Rees N.V., Zhou Y.G., Compton R.G. (2011). The aggregation of silver nanoparticles in aqueous solution investigated via anodic particle coulometry. ChemPhysChem.

[9] Zhou X., Xu W., Liu G., Panda D., Chen P. (2010). Size-dependent catalytic activity and dynamics of gold nanoparticles at the single-molecule level. J. Am. Chem. Soc..

[10] Vidal-Iglesias F.J., Solla-Gullón J., Rodrıguez P., Herrero E., Montiel V., Feliu J.M., Aldaz A. (2004). Shape-dependent electrocatalysis: Ammonia oxidation on platinum nanoparticles with preferential (100) surfaces. Electrochem. Commun..

[11] Heyrovsky M., Jirkovsky J. (1995). Polarography and voltammetry of ultrasmall colloids: Introduction to a new field. Langmuir.

[12] Quinn B.M., van’t Hof P.G., Lemay S.G. (2004). Time-resolved electrochemical detection of discrete adsorption events. J. Am. Chem. Soc..

[13] Xiao X., Bard A.J. (2007). Observing single nanoparticle collisions at an ultramicroelectrode by electrocatalytic amplification. J. Am. Chem. Soc..

[14] Xiao X., Fan F.R.F., Zhou J., Bard A.J. (2008). Current transients in single nanoparticle collision events. J. Am. Chem. Soc..

[15] Kwon S.J., Fan F.R.F., Bard A.J. (2010). Observing iridium oxide (IrO_x_) single nanoparticle collisions at ultramicroelectrodes. J. Am. Chem. Soc..

[16] Zhou Y.G., Rees N.V., Compton R.G. (2011). The electrochemical detection and characterization of silver nanoparticles in aqueous solution. Angew. Chem. Int. Ed..

[17] Haddou B., Rees N.V., Compton R.G. (2012). Nanoparticle–electrode impacts: The oxidation of copper nanoparticles has slow kinetics. Phys. Chem. Chem. Phys..

[18] Plowman B.J., Young N.P., Batchelor-McAuley C., Compton R.G. (2016). Nanorod aspect ratios determined by the nano-impact technique. Angew. Chem..

[19] Kwon S.J., Zhou H., Fan F.R.F., Vorobyev V., Zhang B., Bard A.J. (2011). Stochastic electrochemistry with electrocatalytic nanoparticles at inert ultramicroelectrodes—theory and experiments. Phys. Chem. Chem. Phys..

[20] Boika A., Thorgaard S.N., Bard A.J. (2013). Monitoring the electrophoretic migration and adsorption of single insulating nanoparticles at ultramicroelectrodes. J. Phys. Chem. B..

[21] Zhou H., Fan F.R.F., Bard A.J. (2010). Observation of discrete au nanoparticle collisions by electrocatalytic amplification using Pt ultramicroelectrode surface modification. J. Phys. Chem. Lett..

[22] Choi Y.D., Jung S.Y., Kim K.J., Kwon S.J. (2016). Combined blip and staircase response of ascorbic acid-stabilized copper single nanoparticle collision by electrocatalytic glucose oxidation. Asian J. Chem..

[23] Fernando A., Parajuli F., Alpuche-Aviles M.A. (2013). Observation of individual semiconducting nanoparticle collisions by stochastic photoelectrochemical currents. J. Am. Chem. Soc..

[24] Kim B.K., Kim J., Bard A.J. (2015). Electrochemistry of a single attoliter emulsion droplet in collisions. J. Am. Chem. Soc..

[25] Kim B.K., Boika A., Kim J., Dick J.E., Bard A.J. (2014). Characterizing emulsions by observation of single droplet collisions—Attoliter electrochemical reactors. J. Am. Chem. Soc..

[26] Dick J.E., Hilterbrand A.T., Boika A., Upton J.W., Bard A.J. (2015). Electrochemical detection of a single cytomegalovirus at an ultramicroelectrode and its antibody anchoring. Proc. Natl. Acad. Sci. USA.

[27] Dick J.E., Renault C., Bard A.J. (2015). Observation of single-protein and DNA macromolecule collisions on ultramicroelectrodes. J. Am. Chem. Soc..

[28] Sobolev A., Valkov A., Kossenko A., Wolicki I., Zinigrad M., Borodianskiy K. (2019). Bioactive coating on Ti alloy with high osseointegration and antibacterial Ag nanoparticles. ACS Appl. Mater. Interfaces.

[29] Li H., Sun Z., Zhong W., Hao N., Xu D., Chen H.Y. (2010). Ultrasensitive electrochemical detection for DNA arrays based on silver nanoparticle aggregates. Anal. Chem..

[30] Hao R., Fan Y., Zhang B. (2016). Electrochemical detection of nanoparticle collision by reduction of silver chloride. J. Electrochem. Soc..

[31] Defnet P.A., Zhang B. (2021). Collision, Adhesion, and Oxidation of Single Ag Nanoparticles on a Polysulfide-Modified Microelectrode. J. Am. Chem. Soc..

[32] Saw E.N., Kanokkanchana K., Amin H.M., Tschulik K. (2022). Unravelling Anion Solvation in Water-Alcohol Mixtures by Single Entity Electrochemistry. ChemElectroChem.

[33] Mun S.K., Lee S., Kim D.Y., Kwon S.J. (2017). Various current responses of single silver nanoparticle collisions on a gold ultramicroelectrode depending on the collision conditions. Asian J. Chem..

[34] Liu R., Shen X., Wang D. (2021). Electrochemical Collision of Single Silver Nanoparticles in Carbon Nanopipettes. Anal. Chem..

[35] Sikes J.C., Niyonshuti I.I., Kanokkanchana K., Chen J., Tschulik K., Fritsch I. (2022). Single particle electrochemical oxidation of polyvinylpyrrolidone-capped silver nanospheres, nanocubes, and nanoplates in potassium nitrate and potassium hydroxide solutions. J. Electrochem. Soc..

[36] Abd El Rehim S.S., Hassan H.H., Ibrahim M.A., Amin M.A. (1998). Electrochemical behaviour of a silver electrode in NaOH solutions. Monatsh. Chem..

[37] Fernando I., Zhou Y. (2019). Impact of pH on the stability, dissolution and aggregation kinetics of silver nanoparticles. Chemosphere.

[38] Jung S.Y., Joo J.W., Kwon S.J. (2016). Observation of blip response in a single Pt nanoparticle collision on a Cu ultramicroelectrode. Bull. Korean Chem. Soc..

[39] Choi Y.S., Jung S.Y., Joo J.W., Kwon S.J. (2014). Observation of electrocatalytic amplification of iridium oxide (IrO_x_) single nanoparticle collision on copper ultramicroelectrodes. Bull. Korean Chem. Soc..

[40] Gupta A.K., Gupta K.S., Gupta Y.K. (1985). Kinetics and mechanism of the uncatalyzed and silver (I)-catalyzed oxidation of hydrazine with peroxodiphosphate in acetate buffers. Inorg. Chem..

[41] Clark A.J., Pickering W.F. (1967). The silver (II) oxide-hydrazine reaction. J. Inorg. Nucl..

[42] Fu Q., Wagner T. (2007). Interaction of nanostructured metal overlayers with oxide surfaces. Surf. Sci. Rep..

[43] Park J.H., Boika A., Park H.S., Lee H.C., Bard A.J. (2013). Single collision events of conductive nanoparticles driven by migration. J. Phys. Chem. C.

[44] Park J.H., Thorgaard S.N., Zhang B., Bard A.J. (2013). Single particle detection by area amplification: Single wall carbon nanotube attachment to a nanoelectrode. J. Am. Chem. Soc..

[45] Oja S.M., Robinson D.A., Vitti N.J., Edwards M.A., Liu Y., White H.S., Zhang B. (2017). Observation of multipeak collision behavior during the electro-oxidation of single Ag nanoparticles. J. Am. Chem. Soc..

[46] Bard A.J., Faulkner L.R. (2001). Electrochemical Methods, Fundamentals and Applications.

[47] Pillai Z.S., Kamat P.V. (2004). What factors control the size and shape of silver nanoparticles in the citrate ion reduction method. J. Phys. Chem. B.

