# Supplementary material for: Electrochemical Detection and Analysis of Various Current Responses of a Single Ag Nanoparticle Collision in an Alkaline Electrolyte Solution
Source: Int J Mol Sci. 2022 Jul 5;23(13):7472. doi: 10.3390/ijms23137472 (PMC9267213; doi:10.3390/ijms23137472)
Supplement: Supplementary file 1 [file ijms-23-07472-s001.zip › ijms-1741050-supplementary.pdf]

Supporting Information for:

Electrochemical Detection and Analysis of Various Current Responses of a Single Ag Nanoparticle Collision in an Alkaline Electrolyte Solution

Ki Jun Kim, and Seong Jung Kwon*

Department of Chemistry, Konkuk University, 120 Neungdong-ro Gwangjin-gu Seoul 05029, Republic of Korea; sjkwon@konkuk.ac.kr

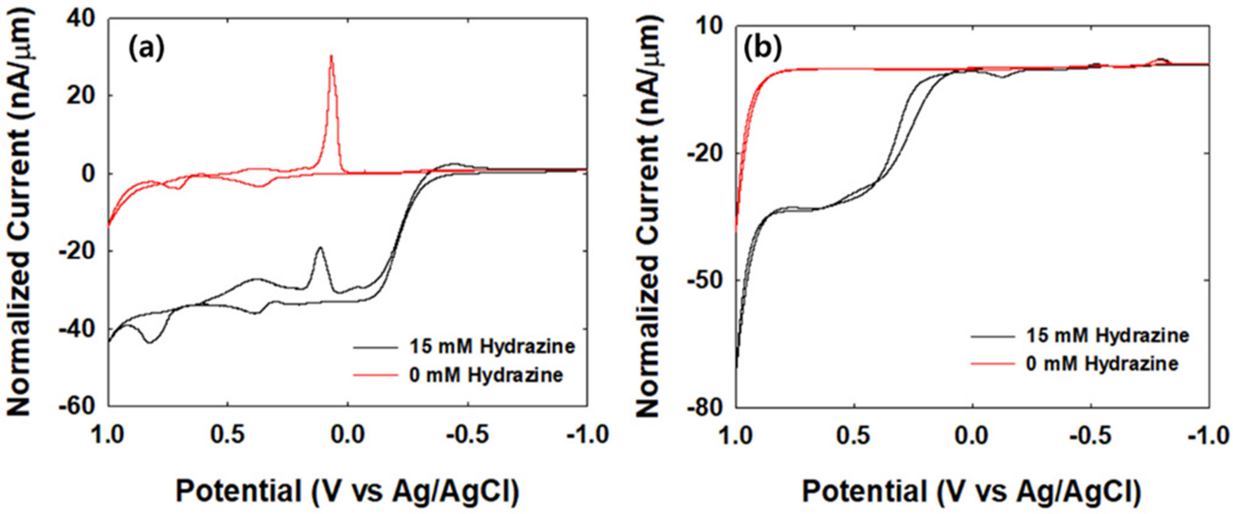

Figure S1. Cyclic voltammograms of (a) Ag UME and (b) Cu UME in 0.1 M NaOH solution with/without 15 mM hydrazine. Scan rate is 0.1 V/s.

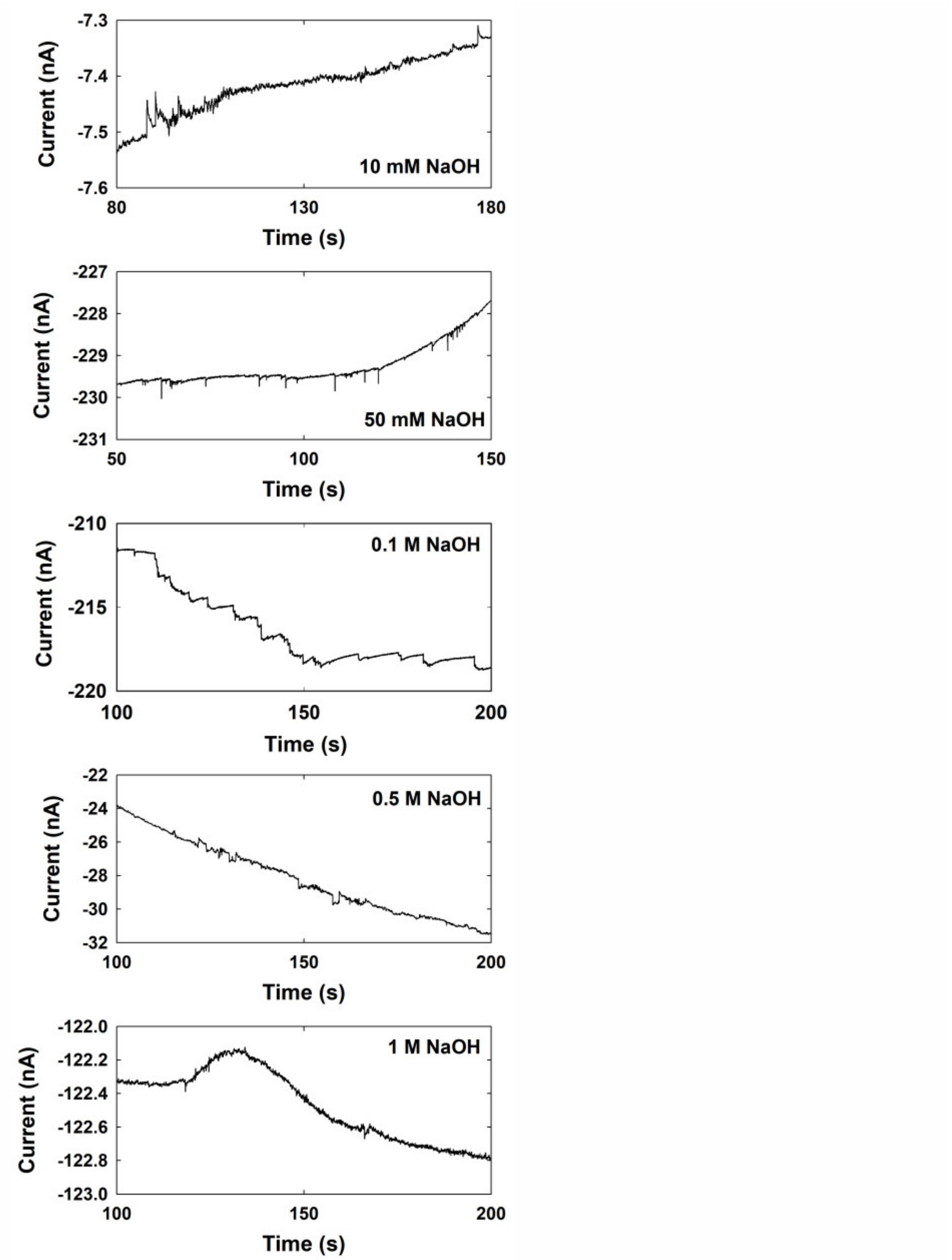

Figure S2. Chronoamperometric curves for single Ag NP collisions at the Cu UME with applied potentials 0.7 V in a various NaOH concentration form 10 mM to 1 M containing 15 mM hydrazine. Data acquisition time is 50 ms.

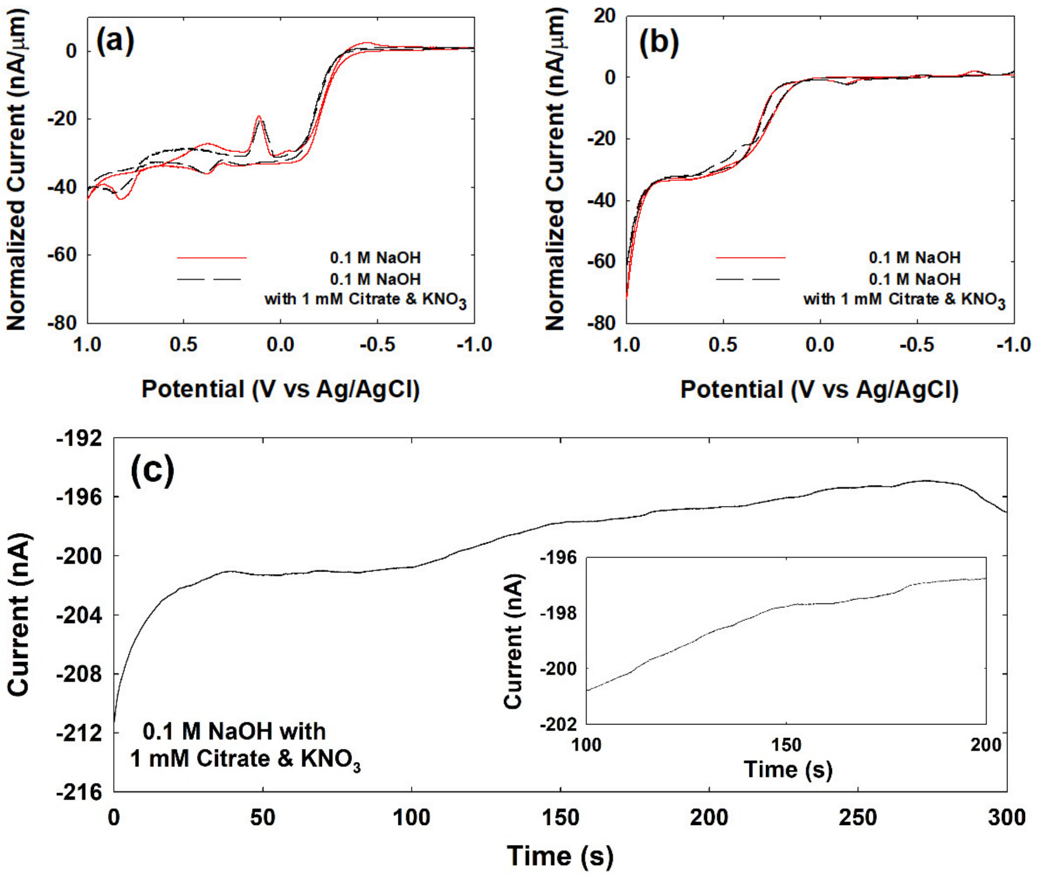

Figure S3. CVs for (a) Ag UME (b) Cu UME in 0.1 M NaOH containing 15 mM hydrazine and with(black)/without(red) 1 mM citrate and KNO_3. (c) Chronoamperometric curve at 0.7 V applied Cu UME in 0.1 M NaOH containing 15 mM hydrazine injected by 1 mM citrate and KNO_3 solution instead of Ag NP.

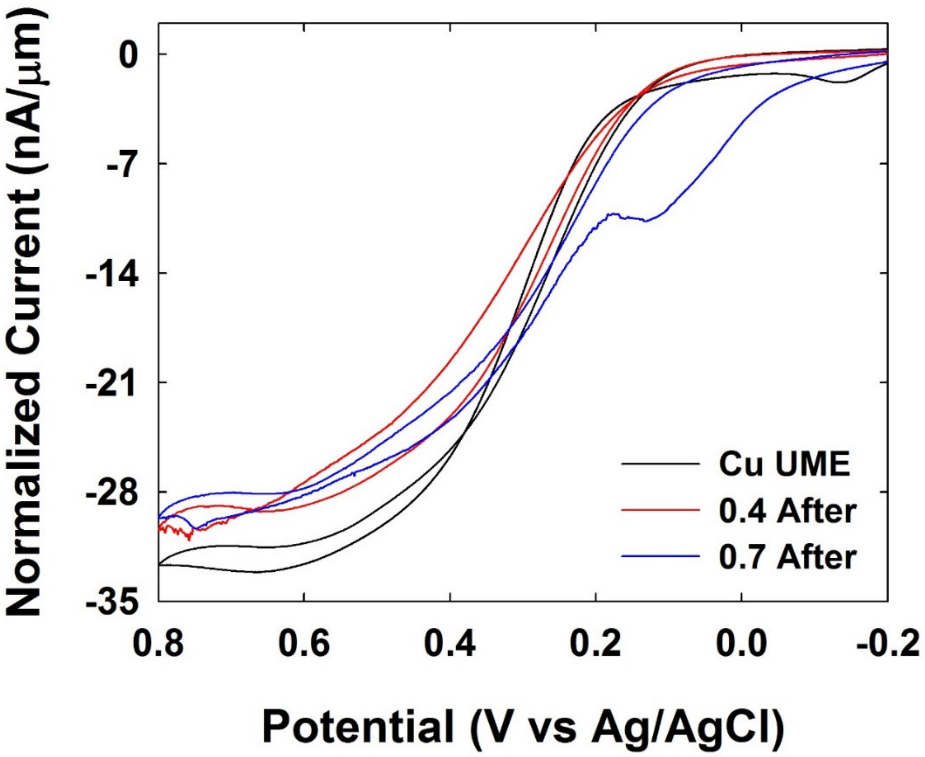

Figure S4. CVs of Cu UME after the 1000 sec of chronoamperometric measurement of single Ag NP collision at 0.4 V (red) or 0.7 V (blue) in 0.1 M NaOH solution with 15 mM hydrazine. The background of Cu UME (black) was obtained in 0.1 M NaOH solution with 15 mM hydrazine without Ag NP collision. Scan rate is 0.1 V/s.

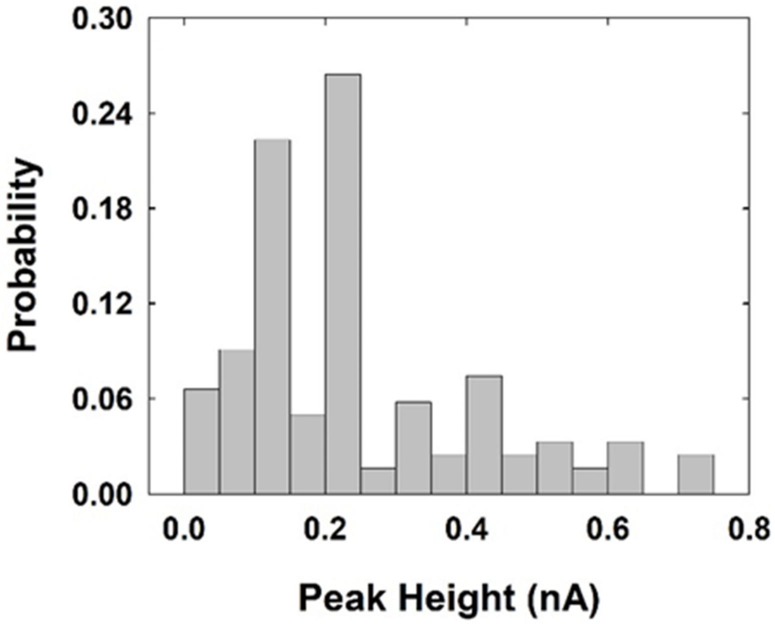

Figure S5. Size distribution of peak height of single Ag NP collision signal at the 0.7 V of applied potential. Average size of peak height is 0.23 ± 0.16 nA.

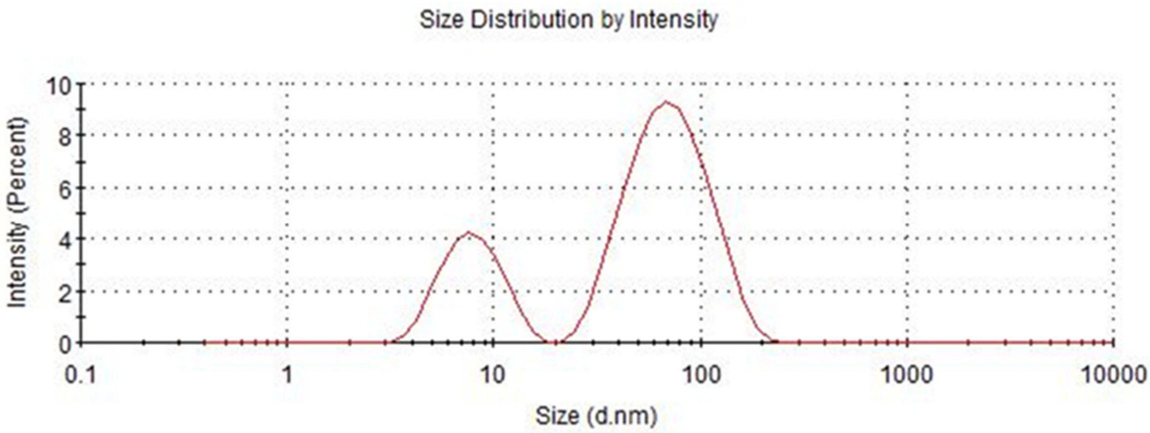

Figure S6. Size distribution of Ag NP by Dynamic Light Scattering (DLS). Average diameter is 59 ± 25 nm.

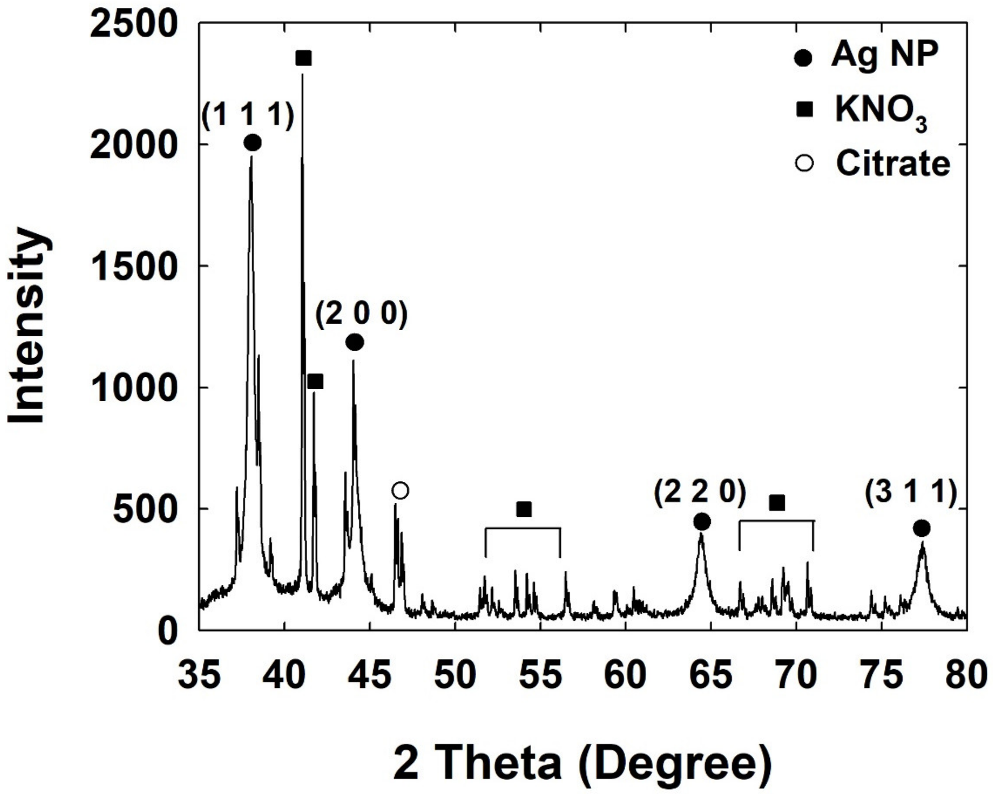

Figure S7. XRD data for synthesized Ag NP.

For measuring XRD about synthesized Ag NP, salting out method with sufficient KNO_3 was used for precipitating Ag NP. As shown figure S2, peaks for Ag (111), (200), (220), and (311) facets were observed. Some of residual peaks were originated from KNO_3 and citrate which were salt and capping agent.
